# Enhanced Migration of Fuchs Corneal Endothelial Cells by Rho Kinase Inhibition: A Novel Ex Vivo Descemet’s Stripping Only Model

**DOI:** 10.3390/cells13141218

**Published:** 2024-07-19

**Authors:** Mohit Parekh, Annie Miall, Ashley Chou, Lara Buhl, Neha Deshpande, Marianne O. Price, Francis W. Price, Ula V. Jurkunas

**Affiliations:** 1 Schepens Eye Research Institute of Mass Eye and Ear, Department of Ophthalmology, Harvard Medical School, 20 Staniford Street, Boston, MA 02114, USA; mnparekh@meei.harvard.edu (M.P.);; 2 Faculty of Arts and Sciences, Harvard College, Boston, MA 02138, USA; 3 Wellman Center for Photomedicine, Massachusetts General Hospital, Boston, MA 02114, USA; 4 Cornea Research Foundation of America, Indianapolis, IN 46260, USA; 5 Cornea and Refractive Surgery Service, Massachusetts Eye and Ear Infirmary, 243 Charles Street, Boston, MA 02114, USA

**Keywords:** cornea, Fuchs endothelial corneal dystrophy, corneal endothelium, cells, migration, ROCK inhibitor, Descemet’s Stripping Only

## Abstract

Descemet’s Stripping Only (DSO) is a surgical technique that utilizes the peripheral corneal endothelial cell (CEnC) migration for wound closure. Ripasudil, a Rho-associated protein kinase (ROCK) inhibitor, has shown potential in DSO treatment; however, its mechanism in promoting CEnC migration remains unclear. We observed that ripasudil-treated immortalized normal and Fuchs endothelial corneal dystrophy (FECD) cells exhibited significantly enhanced migration and wound healing, particularly effective in FECD cells. Ripasudil upregulated mRNA expression of Snail Family Transcriptional Repressor (*SNAI1/2*) and Vimentin (*VIM*) while decreasing Cadherin (*CDH1*), indicating endothelial-to-mesenchymal transition (EMT) activation. Ripasudil activated Rac1, driving the actin-related protein complex (ARPC2) to the leading edge, facilitating enhanced migration. Ex vivo studies on cadaveric and FECD Descemet’s membrane (DM) showed increased migration and proliferation of CEnCs after ripasudil treatment. An ex vivo DSO model demonstrated enhanced migration from the DM to the stroma with ripasudil. Coating small incision lenticule extraction (SMILE) tissues with an FNC coating mix and treating the cells in conjunction with ripasudil further improved migration and resulted in a monolayer formation, as detected by the ZO-1 junctional marker, thereby leading to the reduction in EMT. In conclusion, ripasudil effectively enhanced cellular migration, particularly in a novel ex vivo DSO model, when the stromal microenvironment was modulated. This suggests ripasudil as a promising adjuvant for DSO treatment, highlighting its potential clinical significance.

## 1. Introduction

The human cornea serves as the outermost transparent layer of the eye, playing a pivotal role in refracting incident light from the surrounding. The preservation of corneal transparency is vital for ensuring clear vision, and this is predominantly regulated by the posterior hexagonal monolayer of endothelial cells situated at the corneal–aqueous humor interface. Human corneal endothelial cells (HCEnCs) are important for maintaining corneal deturgescence, which is carried out via the active pumping mechanism and barrier function [1]. It is essential to preserve the hydration status of the cornea for maintaining tissue transparency. Dysfunctional or diseased HCEnCs can result in the over-accumulation and retention of excessive fluid in the cornea, leading to edema and loss of vision, hence the maintenance of these cells is crucial.

Although humans are born with a significant amount of HCEnCs, these cells decrease at an average rate of 0.6% per year from birth to adulthood [2,3,4,5]. In addition, several factors such as age, trauma, ocular surgeries, or dystrophies can lead to temporary or irreversible corneal blindness. Fuchs endothelial corneal dystrophy (FECD) is a complex, slow-progressing, genetically associated, age-related disorder which is predominant in females [6,7]. FECD is generally characterized by a slow decline of corneal endothelial cells that leads to polymorphism and abnormal extracellular matrix (ECM) deposition called guttae [8,9,10]. It is one of the most common indications for corneal transplantation [11], which is the only treatment available with no pharmacologic alternatives. The most favorable option to restore the vision in such case is a corneal replacement by transplanting a cadaveric donor graft [12]. However, it is challenging to rely on the availability of the human donor corneas, which unfortunately is limited [11]. Although several attempts have been made to increase the utilization of each donor cornea, the corneal tissue availability remains a global concern. Alternative approaches to culture HCEnCs in vitro using inducers that aid in proliferation have also been investigated [13,14,15]; however, these techniques also partially rely on healthy cadaveric donor endothelial cells as a source.

Descemet’s Stripping Only (DSO) was therefore introduced to partially reduce the reliance on the donor tissues for the treatment of endothelial dysfunction [16]. Being a relatively new technique, DSO has been clinically evaluated only by a small number of groups [17,18,19,20]. Based on the early work by Okumura et al. [21] and Kinoshita et al. [15], the utility of Rho-associated protein kinase-inhibitor (ROCKi) in enhancing HCEC migration after DSO was first described by Moloney [16], and subsequently has been investigated in several other clinical studies [18,19,20]. In one study, 10 FECD patients underwent DSO with immediate netarsudil instillation in one eye and delayed netarsudil in the other; the former group showed a reduction in corneal clearance time compared to the delayed netarsudil use [19]. Macsai et al. demonstrated that patients treated with DSO and topical ripasudil (0.4%) recovered vision faster and achieved significantly higher average endothelial cell counts compared to those treated with DSO alone [20]. Eyes treated with DSO alone recovered vision in 6.5 weeks, while those treated with DSO and ripasudil together significantly reduced the rehabilitation time to 4.6 weeks. These isolated case reports suggest that the use of ROCKi in conjunction with DSO could serve as an alternative treatment for FECD [18,19,20]. Despite promising early post-operative outcomes, the long-term efficacy of the DSO technique remains to be determined. Additionally, the mechanism underlying the predictability of corneal clearance following DSO has not been fully understood.

A successful DSO treatment removes unhealthy central corneal endothelium and Descemet’s membrane (DM) and relies on the centripetal migration of the HCEnCs from the periphery to reconstruct the wounded area in the center. Since HCEnCs have an extremely limited regenerative potential in vivo, cell enlargement and migration are mainly responsible for wound healing, as observed in other cell types [22]. However, the mechanism that regulates the migration of HCEnCs on bare stroma after descemetorhexis in FECD has not been fully studied. In addition, no ex vivo models are available that allow for the investigation of single-cell migration mimicking the DSO technique. Importantly, the predictability factors related to successful corneal endothelial wound healing and surgical outcomes following DSO have not been completely understood.

FECD is strongly associated with CTG repeat expansion in the transcription factor (*TCF4* gene). A study found that predicting the time to corneal clearance based on CTG repeats was not possible, although a significant correlation between allele repeats and the achievement of corneal clearance was observed [23]. In addition, smaller descemetorhexis has been associated with faster recovery, while diabetes appeared to delay recovery after DSO. Visual outcomes for patients undergoing DSO did not seem to be adversely affected by concurrent cataract surgery [24]. Another retrospective study failed to identify significant predictive factors for better outcomes after DSO based on age, pachymetry, and endothelial cell count. Nevertheless, although some authors recommend better outcomes with a smaller central descemetorhexis (4 mm), there is no definitive predictive factor for corneal clearance after DSO [24].

Rho-associated protein kinase is a pleiotropic kinase that has the potential to regulate several cellular functions such as cytoskeletal rearrangement, cell adhesion, migration, cell survival, proliferation, and anti-apoptosis [25,26,27,28,29,30,31,32,33]. In fact, in pilot clinical studies, ROCKi have been applied to reduce the time to corneal clearance after DSO [16,19]. Although initially ROCKi was introduced to enhance the survival of HCEnCs, it was later demonstrated to improve the corneal endothelial cell migration partly via the regulation of lamellipodial dynamics [21,28,34,35]. Inhibiting RhoA GTPase activates Rac1, which, in turn, triggers the WAVE regulatory complex to drive actin-related protein (ARP2/3) complex-mediated actin polymerization, forming lamellipodial structures [36,37]. However, this phenomenon has never been studied in relation to DSO.

In our previous study, we developed a live-cell imaging technique [38] and showed the migration of the cells on the DM of cadaveric donors. However, the effect of ROCKi (ripasudil) on the migration of cells both on the native DM and the stroma (similar to DSO) from normal and FECD donors has never been shown. We therefore aim to understand the underlying mechanism that enhances the cellular migration of FECD cells and apply it on an ex vivo DSO model to investigate the true benefits of using ripasudil for the treatment of FECD. To achieve this, we studied the effect of ripasudil on the random migration (velocity and displacement) of immortalized normal and FECD cells in vitro. Wound healing on immortalized cells was also investigated after treating the cells with ripasudil. Using ex vivo donor tissues, we further investigated whether the FECD cells migrate faster compared to normal cells on their respective DMs when treated with ripasudil. We also developed a novel ex vivo DSO model to evaluate if ripasudil enhances the migration of cells from the DM to the stroma, especially when the ECM microenvironment is modified.

## 2. Methods

### 2.1. Cell Lines and Human Cadaveric Tissues

Immortalized cells were generated by transfecting cells from normal cadaveric donor tissues and FECD patient specimens with simian virus 40 (SV40) [38]. The nomenclature of the immortalized normal cells (SVN1-67F) is as follows: transfection agent (SV40), normal cadaveric donor tissue (N1), age of the donor (67), and gender of the donor (F). The same was followed for the FECD donors (SVF1-73F, SVF5-54F, and SVF3-76M), where N1 was replaced by F1, F5 or F3 (depending on the tissue number) for FECD tissues.

For the ex vivo studies, the normal cadaveric human donor corneo-scleral tissues were obtained from Eversight (Ann Arbor, MI, USA) following a written consent, and the FECD patient specimen (Descemet membrane–endothelium complex) were shipped to our lab by Price Vision Group (Indianapolis, IN, USA) for research use. The study was conducted in accordance with the tenets of the Declaration of Helsinki and was approved by the Massachusetts Eye and Ear Institutional Review Board. The donor and the patient specimen were stored in Optisol-GS (Bausch&Lomb, Bridgewater, NJ, USA) until used. For all the subsequent experiments, the cells were plated on an FNC coating mix base (AthenaES, Baltimore, MD, USA) using the cell culture media (Chen’s) [39,40].

### 2.2. Cell Viability Study

The cells (immortalized normal and FECD) were plated at 8000 cells/0.32 cm^2^ in a cell culture microplate (96-well; Greiner Bio-One North America Inc., Monroe, NC, USA) precoated with FNC for the luminescence assay using CellTiter glo (VWR, Bridgeport, NJ, USA). The cells were cultured for 24 h at 5% CO_2_ and 37 °C. On the following day, the cells were treated with 0.3 µM, 1 µM, and 10 µM of ripasudil (K-321; Kowa Company, Ltd., Nagoya, Japan) dissolved in OptiMEM-I for the experimental condition, or just OptiMEM-I supplemented with the vehicle dimethyl sulfoxide (DMSO) serving as a control (for all subsequent experiments), and incubated for 24 h at 5% CO_2_ and 37 °C. At the end of the 24 h, the plate was removed from the incubator and brought to room temperature (RT) for 10 min. The CellTiter glo was added to the media (1:1) and the plate was incubated at RT in the dark for another 15 min, followed by shaking it at a medium pace on a shaker for 2 min. Luminescence was measured using the Synergy H1 (Agilent Technologies, Inc., Santa Clara, CA, USA) plate reader and the percentage viability was calculated compared to the control.

### 2.3. Cell Toxicity Study

All of the immortalized cells were cultured at 1.5 × 10^5^/3.8 cm^2^ well on an FNC-coated base for live/dead analysis. Following 24 h of treatment with 0.3 µM, 1 µM, and 10 µM of ripasudil dissolved in OptiMEM-I, the untreated control and the treated wells were briefly washed with sterile 1x phosphate-buffered saline (PBS). The cells were then treated with a solution of 1 µM of Hoechst 33342 and 2 µM of Ethidium Homodimer (live/dead assay kit, Thermofisher, Waltham, MA, USA). Leaving the solution in the well, the cells were imaged after 15 min of incubation at RT in the dark using a Leica DMi8 microscope (Leica Microsystems Inc., Deerfield, IL, USA). The pictures were exported to ImageJ and converted to binary images, followed by analyzing the particle counts. The percentage of dead cells was counted by using the number of red (ethidium homodimer + ve) compared to blue (Hoechst + ve) particles.

### 2.4. Scratch Assay—In Vitro Wound Healing

**Figure 1:**
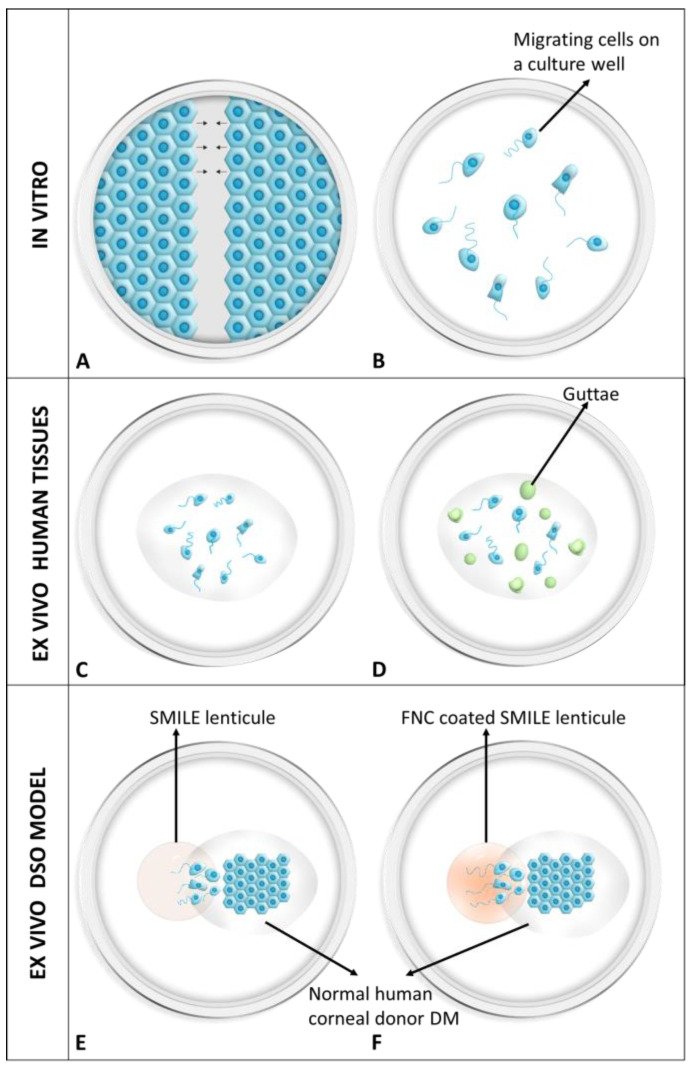
Illustration of different methods used to study migration of human corneal endothelial cells. (**A**) In vitro scratch assay and (**B**) random migration assay using immortalized normal and FECD cells. (**C**) Ex vivo random migration of normal human and (**D**) FECD cells on their respective native Descemet membrane. (**E**) A schematic of the ex vivo DSO model showing the DM attached on the SMILE lenticule without the FNC coat and (**F**) with the FNC coating. DM: Descemet’s membrane; FECD: Fuchs endothelial corneal dystrophy; FNC: fibronectin collagen mix; SMILE: small incision lenticule extraction.

Normal and FECD cell lines were grown to a confluent monolayer in an FNC precoated 12-well culture plate. A linear scratch was performed manually using a standard 10 µL pipette tip (Figure 1A). Wells were washed with OptiMEM-I and supplemented with OptiMEM-I (control) or OptiMEM-I (ripasudil 1 µM). Live-cell imaging was performed using the time-lapse feature of the LASX imaging software version 3.7.4 integrated on the Leica DMi8 microscope, acquiring images at 10× magnification at 6 h intervals over 24 h. Four separate regions of interest were imaged per wound per condition (ripasudil+/−) per cell line, and independent experiments were performed with at least three passages indicative of three biological replicates. The captured images were imported to ImageJ, and using the freehand tool, the wounded area was marked on the image. The wound area was measured in ImageJ by comparing it to the 0 h time point to determine the percentage of wound closure.

### 2.5. Random Migration Study

#### 2.5.1. In Vitro

All the cell lines were cultured at a low plating density, i.e., 5 × 10^5^/3.8 cm^2^, on an FNC-coated base for 24 h in the incubator consisting of 5% CO_2_ maintained at 37 °C (Figure 1B). The nucleus was stained using 1 µM of Hoechst 33342 (ThermoFisher, Waltham, MA, USA) for 30 s at RT followed by a single wash with 1× PBS. The cells were then treated either with OptiMEM-I for the controls or with 1 µM of ripasudil in OptiMEM-I and subjected to live-cell imaging for 6 h, with images captured at an interval of 5 min.

#### 2.5.2. Ex Vivo Tissues

The donor characteristics and sample size for the ex vivo studies are listed in Table 1 and Table 2, respectively. After washing with sterile PBS, the corneo-scleral tissues from the normal human cadaveric donors were placed on a trephine base with the endothelium side facing the air. A standard Descemet membrane endothelial keratoplasty (DMEK) stripping technique was used to peel the Descemet membrane–endothelial complex [41]. The tissue was completely peeled and washed in a sterile 2 mL Eppendorf vial filled with 1x PBS. Tissues from the FECD patients were washed directly in 1x PBS and the subsequent steps were followed for the FECD tissues. The tissues were stained using 1 µM of Hoechst 33342 for 30 s at RT, washed once to remove the excess Hoechst, and stored in serum-free OptiMEM-I. The wells of a 12-well plate were coated with FNC for 30 s at RT. The stained tissue was gently unrolled and flattened in the well with the endothelium facing the air. The tissue rolling outwards was an indication of the correct endothelial side orientation. Once the tissue was unrolled, it was air-dried for 1 min and approximately 400 µL of the serum-free OptiMEM-I with or without ripasudil was added on the tissue. The cells on the DM from normal human cadaveric donors (Figure 1C) and the FECD patient specimen (Figure 1D) were analyzed using live-cell imaging, as described later.

### 2.6. Descemet’s Stripping Only (DSO): An Ex Vivo Model

After peeling the Descemet membrane–endothelial complex, the tissues were stored for less than 6 h in Optisol-GS media. The remaining corneal button was subjected to the small incision lenticule extraction (SMILE) technique to extract approximately 120 µm of stromal lenticule using the VisuMax femtosecond laser (Carl Zeiss Meditec, Dublin, CA, USA). Briefly, the corneal tissue without the endothelium was mounted on a Barron artificial anterior chamber (Katena, Parsippany, NJ, USA) and the tissue was adjusted beneath the laser area. The cap diameter (mm), thickness (µm), and side-cut angle (°) were maintained at 7.2, 120, and 90, respectively, and the lenticule was set at 6, 91, and 90, respectively. The lenticule was dissected using a dissector and removed using forceps. The SMILE lenticule was washed and attached on the base of a pre-FNC-coated 12-well plate after air-drying for approximately 5 min to facilitate adhesion. The peeled Descemet membrane–endothelial complex tissue was stained with Hoechst 33342 (1 µM) for 1 min at RT in the dark, washed thoroughly, and draped onto the SMILE stroma with the endothelium side facing up. The tissue was unrolled completely, ensuring half of the graft was attached on the stroma, and the other half remained on the plastic base precoated with the FNC to ensure the construct did not move during the image acquisition (Figure 1E). FNC coating was also applied on the stroma for a separate set of experiments to investigate whether a base coat facilitates enhanced endothelial cell migration (Figure 1F).

### 2.7. Data Acquisition for the Migration Study

The 12-well tissue culture plate was placed on the Oko-H-301-K-frame (Okolab, Sewickley, PA, USA) with a 12-well plate holder covered by the lid from the same company fixed to the Leica DMi8 fluorescence inverted microscope. The cells were imaged at 10× magnification using a motorized 3-plate stage equipped with an all-in-one stage-top incubator, the UNO-T-Hypremixed (Okolab), with humidity and temperature controllers connected to a premixed 5% CO_2_/95% air tank set at a 15 psi gas flow. Automated image acquisition was carried out every 5 min for a duration of 6 h (in vitro or ex vivo migration) or between day 4 and day 8 (ex vivo DSO study). At least 5 regions of interest/technical replicates were selected per specimen and 3 biological replicates were performed for the analysis. The tissues for the DSO model were stored for up to 60 days, with the replacement of media every alternate day and supplementation with the ripasudil drug every 3 days up to day 30.

The image sequence of the Hoechst-positive cells obtained every 5 min was imported to ImageJ version 1.52 and the scale was removed to create a baseline setting of the scale in pixels. The Trackmate plugin was used to track the cells following the Downsample Log Detector. A blob diameter of 25 pixel for cell lines and 40 pixels for the cells on the ex vivo tissues was selected and maintained for all of the samples. Following the selection of the hyperstack displayer, the data were acquired by mean intensity using a simple lap tracker. The final data on the mean velocity and displacement were obtained from the analysis report generated by ImageJ [42] and subjected to statistical analysis.

### 2.8. RT-PCR

The RNA from the control and ripasudil-treated cells/tissues was extracted using Trizol (Invitrogen, Waltham, MA, USA). The RNA was isolated and purified by washing in absolute propanol and 75% ethanol sequentially and collected in nuclease-free water. The RNA was measured using a NanoDrop (ThermoFisher, Waltham, MA, USA). The iScript cDNA synthesis kit (Bio-Rad, Hercules, CA, USA) was used to reverse-transcribe RNA using a T100 Thermal cycler (Bio-Rad, Hercules, CA, USA) at 25 °C for 5 min, 46 °C for 20 min, and 95 °C for 1 min. RT-PCR was performed by TaqMan gene expression assays (ThermoFisher, Waltham, MA, USA) in an Eppendorf Realplex2 epgradient S mastercycler (Eppendorf, Enfield, CT, USA). Results were normalized to GAPDH internal control. Relative expression was expressed as 2^ΔΔ(−CT)^.

### 2.9. Rac1 Activity Assay

All of the cell lines were plated on a 96-well plate, treated with 1 µM of ripasudil or left untreated as controls, and incubated for 24 h before the GTPase assay was performed using the GTPase-Glo Assay kit (Promega, Madison, WI, USA) and Active Rac1 kit (SignalChem, Richmond, BC, Canada). The solution was prepared with equal volumes of active Rac1 and GTP solution and mixed with the cells for 2 min on an orbital shaker, followed by incubating it at RT for 60 min in dark. GTPase-Glo reagent was then added and incubated at RT for 30 min. After adding the detection reagent, the amount of luminescence was determined using the Synergy H1 96-well plate reader, and the enzyme specific activity was calculated as per the manufacturer’s instructions.

### 2.10. Immunostaining

The ripasudil+/− treated cells/tissues were fixed in 4% paraformaldehyde (PFA) at RT for 20 min and permeabilized with 0.1% triton-x 100 (Sigma-Aldrich, Burlington, MA, USA) at RT for 30 min. The samples were blocked in 2% bovine serum albumin (BSA) (Fisher Scientific, Waltham, MA, USA) for 1 h at RT, followed by incubating in the primary antibodies anti-Ki67 (1:200; Santa Cruz Biotechnology, Inc., Dallas, TX, USA), anti-ZO-1 (1:150; Thermofisher, Waltham, MA, USA), or anti-p34-Arc/ARPC2 (1:50; Millipore, Burlington, MA, USA) stored at 4 °C overnight on a shaker. Secondary antibodies, Alexa fluor 488/594 anti-mouse/-rabbit (Thermofisher, Waltham, MA, USA), respectively, were added on the following day, and the samples were incubated for 1 h at RT in the dark. For the ARPC2 staining, the samples were stained with rhodamine phalloidin (Cytoskeleton, Inc., Denver, CO, USA) before mounting. All of the samples were mounted with VectaSheild containing 4′,6-diamidino-2-phenylindole (DAPI, Vector Laboratories, Inc., Newark, CA, USA). The image acquisition was carried out using the LASX in-built software in the Leica DMi8 microscope. For the ARPC2 staining, the cells were treated with 100 ng/mL of epidermal growth factor for 1 min at RT before fixing. The images were exported to ImageJ. The channels were split and subjected to image threshold and counted using ‘analysis particles’. The total number of green (Ki-67 + ve) to total number of blue (nuclei, Hoechst + ve) percentage was calculated and used for the statistical analysis. For ARPC2, the actin was found at the leading edge and the ARPC2 migrated to the leading edge following lamellipodia formation. Hence, the ARPC2 was correlated to actin and analyzed using Pearson’s correlation. The R-value was determined using the Coloc2 plugin in ImageJ software version 1.52.

### 2.11. Statistical Analysis

Two-tailed non-parametric Mann–Whitney test with a 95% confidence interval (CI) was used for comparative analysis between the normal and FECD tissues. One-way analysis of variance (Anova) was used to identify the statistical difference between multiple cell lines or those treated with or without ripasudil or FNC+/−. A post hoc Bonferroni test with a 95% CI was used for multiple comparisons, assuming the homogeneity of variance across comparative groups. The data were analyzed using GraphPad Prism version 7 with the probability value of less than 0.05 being considered statistically significantly different. All of the in vitro experiments were repeated at least thrice using three technical replicates, and the ex vivo experiments were performed using at least three biological replicates.

## 3. Results

### 3.1. Ripasudil (1 µM) Does Not Induce Cell Toxicity in Normal and FECD Cells

In our initial investigations, a dose–response study was conducted to examine the influence of ripasudil, administered at three different concentrations (0.3 µM, 1 µM, and 10 µM), on cellular viability and mortality. The viability assessment by the luminescent method did not show a significant loss of viability in SVN1-67F (96 ± 6%), SVF1-73F (91 ± 2%), SVF3-76M (97 ± 14%), and SVF5-54F (95 ± 2%) cells when treated with 1 µM of ripasudil compared to their respective untreated controls (Supplementary Figure S1A). Utilizing ethidium homodimer to evaluate cell mortality, a similar trend in cell death was observed in SVN1-67F (5 ± 2%), SVF1-73F (4 ± 1%), SVF3-76M (7 ± 1%), and SVF5-54F (3 ± 0.5%) cells. Notably, no cell death was observed in these cells when treated with 1 µM of ripasudil compared to their corresponding untreated controls; therefore, the 1 µM concentration was utilized in further experiments (Supplementary Figure S1B).

### 3.2. Trackmate Analysis Shows Increased Random Cell Migration in FECD

**Figure 2:**
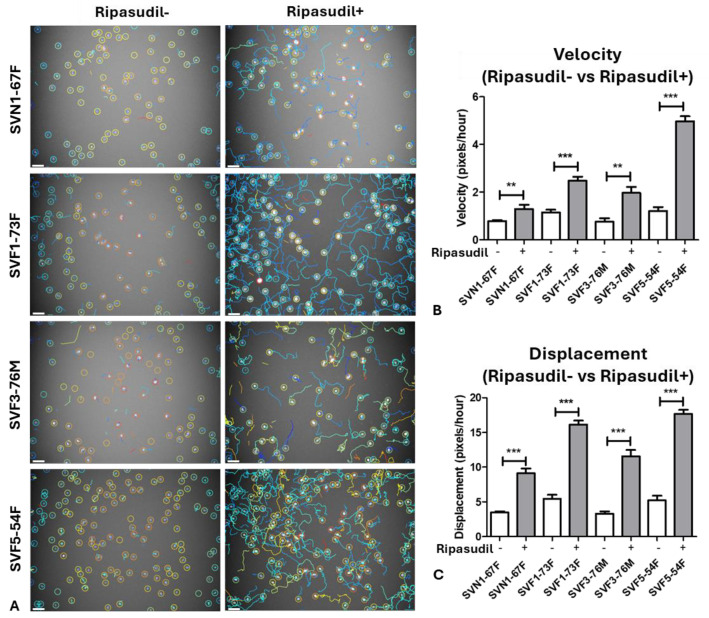
Migration (velocity and displacement) analysis of normal and FECD-immortalized cells with and without ripasudil. (**A**) Trackmate analysis using ImageJ showing the migration of immortalized cells with and without ripasudil. Graphs representing (**B**) velocity (pixels/hour) and (**C**) displacement (pixels/hour) of normal and FECD cell lines with ripasudil treatment compared to their respective untreated controls. ** *p* < 0.01; *** *p* < 0.001. The data are expressed as mean ± SEM. Scale: 50µm.

First, we compared the individual cell migration between the normal and FECD cell lines by quantifying the velocity (speed) and displacement (distance covered) of a single cell movement from point A to B using Trackmate analysis (Figure 2A). We observed that the velocities (pixels/hour) of the normal SVN1-67F (0.8 ± 4.2) cells were lower (*p* < 0.01) compared to FECD SVF1-73F (1.1 ± 3.7) and SVF5-54F (1.2 ± 6.5) cells at baseline, with no difference observed from FECD SVF3-76M (0.8 ± 7.1) cells (Supplementary Figure S2A). Similarly, SVN1-67F (3.4 ± 8.49) cells showed a significantly lower (*p* < 0.01) displacement (pixels/hour) compared to SVF1-73F (5.4 ± 3.7) and SVF5-54F (5.2 ± 7.9), with no difference observed from SVF3-76M (3.3 ± 8.5) cells (Supplementary Figure S2B), indicating that, although the FECD donors exhibit a higher migration capacity at baseline in general, it is not consistent among all FECD cell lines.

However, supplementation with ripasudil (1 µM) significantly enhanced the velocity (pixels/hour) of SVN1-67F (1.3 ± 7.6; *p* < 0.01), SVF1-73F (2.5 ± 2.5; *p* < 0.001), SVF3-76M (2.1 ± 9.9; *p* < 0.01), and SVF5-54F (4.9 ± 2.7; *p* < 0.001) cells, indicating that the drug accelerates migration in all cell lines (Figure 2B). Interestingly, there was a greater increase in the velocity in response to ripasudil in SVF1-73F (2.2-fold; *p* < 0.05), SVF3-76M (2.6-fold; *p* < 0.01), and SVF5-54F (4.1-fold; *p* < 0.001) compared to SVN1-67F (1.6-fold), indicating that FECD cells were more responsive to ripasudil (Supplementary Figure S2C). Concurrently, ripasudil increased the displacement (pixels/hour) of SVN1-67F (9.1 ± 2.2), SVF1-73F (16.1 ± 9.4), SVF3-76M (11.6 ± 2.4), and SVF5-54F (17.7 ± 3.3) compared to the baseline (*p* < 0.001) (Figure 2C). Similarly to velocity, SVF3-76M (3.5-fold; *p* < 0.01) and SVF5-54F (3.4-fold; *p* < 0.01) showed a significantly greater increase in displacement after the ripasudil treatment compared to SVN1-67F cells (2.6-fold) (Supplementary Figure S2D).

### 3.3. Ripasudil Causes Increased Wound Healing in FECD: The Scratch Assay

**Figure 3:**
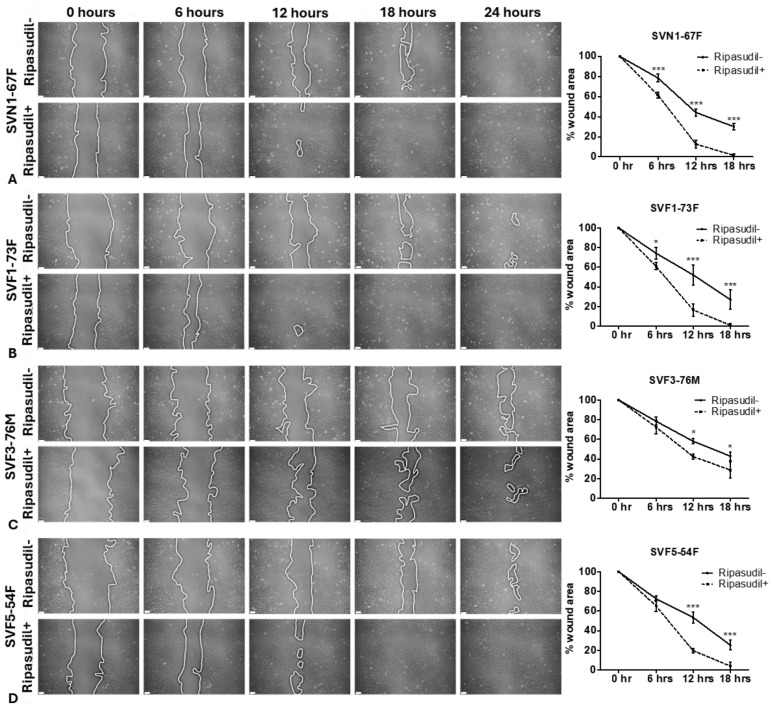
Wound healing analysis of normal and FECD-immortalized cells with and without ripasudil. Wound healing (%) by scratch assay of (**A**) normal (SVN1-67F) and FECD cells (**B**) SVF1-73F, (**C**) SVF3-76M, and (**D**) SVF5-54F with and without ripasudil treatment at different time points. * *p* < 0.05; *** *p* < 0.001. Scale: 50µm.

To investigate the difference in wound healing, a scratch assay was performed, and the percentage of wound healing (area healed after creating a wound) was calculated. Briefly, the cells were cultured to a confluent monolayer and a wound was created to assess the rate of wound healing after the ripasudil treatment. An improved wound healing was observed in the normal (SVN1-67F; *p* < 0.001; Figure 3A) and FECD cells (SVF1-73F; *p* < 0.05; Figure 3B) as early as 6 h after treating the cells with ripasudil. However, the FECD cells SVF3-76M (*p* < 0.05; Figure 3C) and SVF5-54F (*p* < 0.001; Figure 3D) showed a significant improvement in wound healing by 12 h. A complete wound healing was observed in SVN1-67F, SVF1-73F, and SVF5-54F cells, but not in SVF3-76M cells, within 18 h of ripasudil treatment. A similar trend was observed at baseline (without ripasudil treatment), where SVF3-76M cells showed slower wound healing compared to SVN1-67F and FECD cell lines at 18 h (*p* < 0.01) and 24 h (*p* < 0.001) (Supplementary Figure S3), suggesting that SVF3-76M cells are slower compared to the other cells, indicating a heterogeneity in the FECD cells.

### 3.4. Cells Undergo Initial Endothelial-to-Mesenchymal Transition (EMT) after Ripasudil Treatment

**Figure 4:**
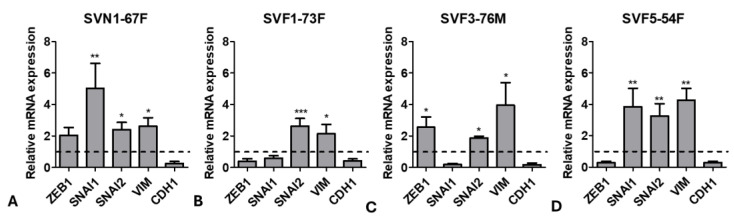
Endothelial-to-mesenchymal transition (EMT) analysis after ripasudil treatment. Upregulation of EMT markers *ZEB1*, *SNAI1*/2, and *VIM*, and the downregulation of *CDH1* in (**A**) normal (SVN1-67F) and FECD (**B**) SVF1-73F, (**C**) SVF3-76M, and (**D**) SVF5-54F cells after treatment with ripasudil, normalized to their respective untreated controls. * *p* < 0.05; ** *p* < 0.01; *** *p* < 0.001. The data are expressed as mean ± SEM.

To investigate the mechanism of enhanced cellular migration, EMT marker expression was examined in FECD and normal cells after the ripasudil treatment. Normal cells (SVN1-67F) showed a significant upregulation in Snail Family Transcriptional Repressor (*SNAI1*) (5-fold; *p* < 0.001), *SNAI2* (2-fold; *p* < 0.05), and Vimentin (*VIM*) (3-fold; *p* < 0.05) after 24 h of ripasudil treatment compared to the untreated controls (Figure 4A). FECD cells (SVF1-73F) showed a significant upregulation of *SNAI2* (3-fold; *p* < 0.001) and *VIM* (2-fold; *p* < 0.05) (Figure 4B), whereas SVF3-76M showed the upregulation of Zinc finger E-box binding homeobox 1 (*ZEB1*) (3-fold; *p* < 0.05), *SNAI2* (2-fold; *p* < 0.05), and *VIM* (4-fold; *p* < 0.05) after ripasudil treatment compared to the untreated controls (Figure 4C). The upregulation of *SNAI1* (4-fold; *p* < 0.01), *SNAI2* (3-fold; *p* < 0.01), and *VIM* (4-fold; *p* < 0.01) was observed in SVF5-54F cells, similarly to what was seen in SVN1-67F (Figure 4D). In all cell lines, ripasudil led to the downregulation of Cadherin (*CDH1*), the gene encoding E-cadherin, required for tight junction formation and known to be decreased during the EMT process.

### 3.5. FECD Cells Migrate Faster Because of Rac1 Activation-Driven Lamellipodia Formation

**Figure 5:**
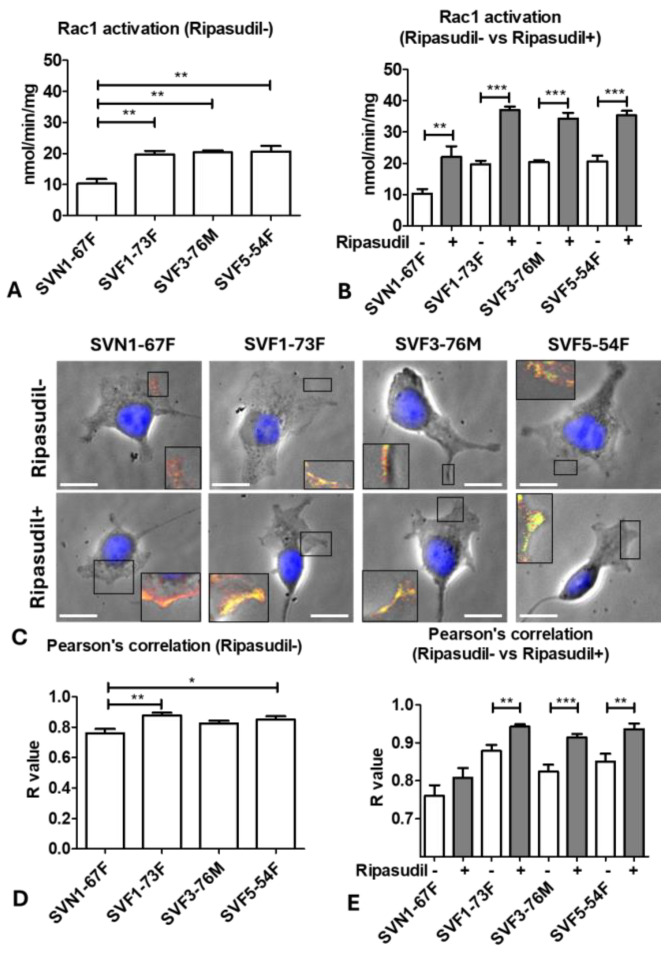
Activation of Rac1 driving lamellipodia formation at the leading edge enhancing cell migration. Rac1 activation in normal and FECD cells at (**A**) baseline and (**B**) after the treatment with ripasudil compared to baseline. (**C**) The expression of ARPC2 at the leading edge in normal and FECD cells with and without ripasudil. Pearson’s correlation between normal and FECD cells at (**D**) baseline and (**E**) after the treatment with ripasudil compared to baseline. * *p* < 0.05; ** *p* < 0.01; *** *p* < 0.001. The data are expressed as mean ± SEM. Blue: nucleus; Red: actin; Green: ARPC2; Yellow: merging of actin and ARPC2. Scale: 10 µm.

Since ripasudil is an inhibitor of RhoA, which is a GTPase with a known function in actin cytoskeleton rearrangements [43], we further investigated the underlying mechanism of how RhoA inhibition enhanced cellular migration during the wound-healing process. Since cellular locomotion is affected by the antagonistic interplay between RhoA and Rac1, which is another Rho-family GTPase involved in actin polymerization, we investigated Rac1 activity by the luminescence assay. Using this technique, we detected that, at baseline, Rac1 activity in the FECD cells SVF1-73F (20 ± 2; *p* < 0.01), SVF3-76M (20 ± 1; *p* < 0.01), and SVF5-54F (21 ± 3; *p* < 0.01) was higher compared to the normal cells (SVN1-67F; 10 ± 3) (Figure 5A), indicating that the intrinsic activation of Rac1 may be associated with the increased migratory capacity of FECD cells at baseline (Supplementary Figure S2). Moreover, ripasudil further caused a greater upregulation of Rac1 in SVF1-73F (37 ± 2; *p* < 0.01), SVF3-76M (34 ± 3; *p* < 0.05), and SVF5-54F (35 ± 3; *p* < 0.05) compared to the normal cells (SVN1-67F; 22 ± 6) (Supplementary Figure S4A), and led to the significant activation of Rac1 compared to the untreated controls in all cell lines: SVN1-67F (2.1-fold; *p* < 0.01), SVF1-73F (1.9-fold; *p* < 0.001), SVF3-76M (1.7-fold; *p* < 0.001), and SVF5-54F (1.7-fold; *p* < 0.001) (Figure 5B).

Rac1 is involved in the cytoskeleton assembly necessary for cell shape maintenance and motility by activating the actin-related protein-2 and 3 (Arp2/3) and causing branched F-actin nucleation [44,45,46]. The growing filaments, known as lamellipodia, are marked by a branched actin filament meshwork formed by the Arp2/3 complex and characterize the leading edge of migrating cells. Lamellipodia produces a protrusive force against the cell membrane, while the whole actin network undergoes retrograde flow due to the contractile force at cell lamella, thus supporting the migration. To investigate this, we utilized ARPC2 staining to mark the leading edge of lamellipodia formation and quantified the colocalization of ARPC2 with actin using Pearson’s correlation value (R-value). The ARPC2 staining was observed at the leading edge of all the cell lines, indicating the migratory potential. The colocalization of ARPC2 with actin (R value) was greater in the FECD cells SVF1-73F (0.88 ± 0.05; *p* < 0.01) and SVF5-54 (0.85 ± 0.07; *p* < 0.05) compared to the normal SNV1-76F (0.76 ± 0.09) cells at baseline (Figure 5C,D). However, the FECD cells SVF1-73F (0.94 ± 0.02; *p* < 0.001), SVF3-76M (0.91 ± 0.03; *p* < 0.001), and SVF5-54F (0.94 ± 0.05; *p* < 0.001) had a greater increase in colocalization compared to SVN1-76F cells (0.81 ± 0.08) after the ripasudil treatment (Supplementary Figure S4B). The FECD cells SVF1-73F (1.07-fold; *p* < 0.01), SVF3-76M (1.11-fold; *p* < 0.001), and SVF5-54F (1.10-fold; *p* < 0.01) showed an increased colocalization after the ripasudil treatment compared to their respective untreated controls, with no difference observed from the normal SVN1-76F (1.06-fold) cells (Figure 5E). This indicated that lamellipodial formation and concurrent remodeling of the actin-network were the driving forces in the promigratory function of ripasudil in FECD.

### 3.6. FECD Cells Migrate Faster on the Native Descemet’s Membrane of the Donor Tissues Ex Vivo

**Figure 6:**
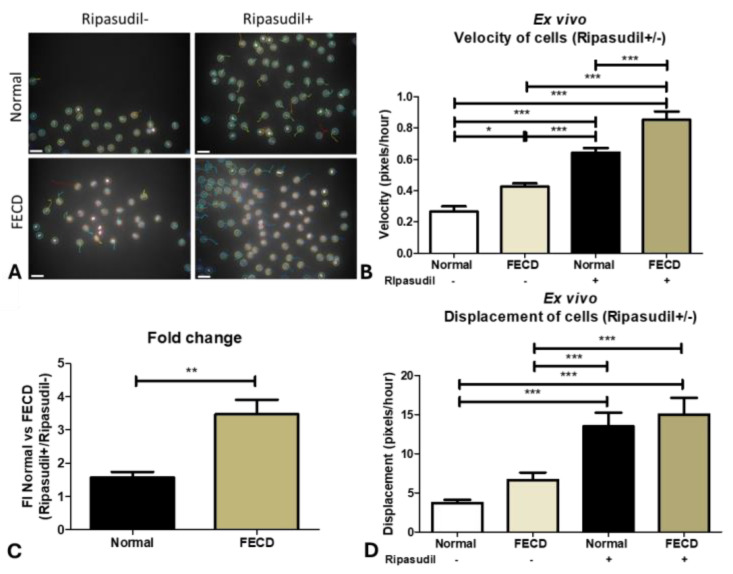
Ex vivo normal and FECD cell migration on their respective Descemet membranes after ripasudil treatment. (**A**) Trackmate analysis of normal and FECD cells using ImageJ showing the migration of cells with and without ripasudil on their respective DMs [the black space represents Descemet’s membrane void of endothelial cells, and the circles mark the border of the nucleus stained with Hoechst]. (**B**) The velocity of normal and FECD cells at baseline and after the treatment with ripasudil ex vivo. (**C**) The fold increase in the velocity of normal and FECD cells after the treatment with ripasudil. (**D**) The displacement of normal and FECD cells at baseline and after the treatment with ripasudil ex vivo. * *p* < 0.05; ** *p* < 0.01; *** *p* < 0.001. The data are expressed as mean ± SEM. Scale: 50 µm.

Since in vivo cellular migration is influenced by the microenvironment of the cellular matrix, next we sought to investigate the random migration of HCEnCs on their native Descemet’s membrane (DM). The cells from the normal human cadaveric donor tissues were exposed to brief trypsin digestion to break the tight junctions, enabling the study of the migratory function. FECD specimens have scattered cells or islands of endothelial cells already in the EMT state, with much lower endothelial cell counts. Therefore, we did not perform trypsin digestion to avoid cell loss. The tissues were stored with or without ripasudil, and the velocity and displacement of the individual cells were analyzed using Trackmate plugin (Figure 6A) after 24 h of incubation for 6 h, with images acquired at an interval of 5 min. We detected that the FECD cells (0.4 ± 0.1; *p* < 0.05) migrated at a higher velocity (pixels/hour) compared to the normal cells (0.3 ± 0.2) on their respective DMs (Figure 6B). However, an enhanced cell velocity was observed on the normal (0.6 ± 0.2; *p* < 0.001) and the FECD (0.8 ± 0.4; *p* < 0.001) DMs after treatment with ripasudil compared to the untreated controls (Figure 6B). Ripasudil treatment caused a greater increase in the velocity of the FECD cells (3.5-fold) compared to the normal cells (1.5-fold) (*p* < 0.001) (Figure 6C). Although no difference in cell displacement was seen between the normal (3.7 ± 2.5) and FECD (6.6 ± 5.7) cells at baseline, ripasudil increased displacement in both normal (13.5 ± 10.3; *p* < 0.001) and FECD cells (15.0 ± 12.5; *p* < 0.001) compared to the untreated controls (Figure 6D).

### 3.7. FECD Tissues Show EMT and Endothelial Cell Proliferation after Ripasudil Treatment

**Figure 7:**
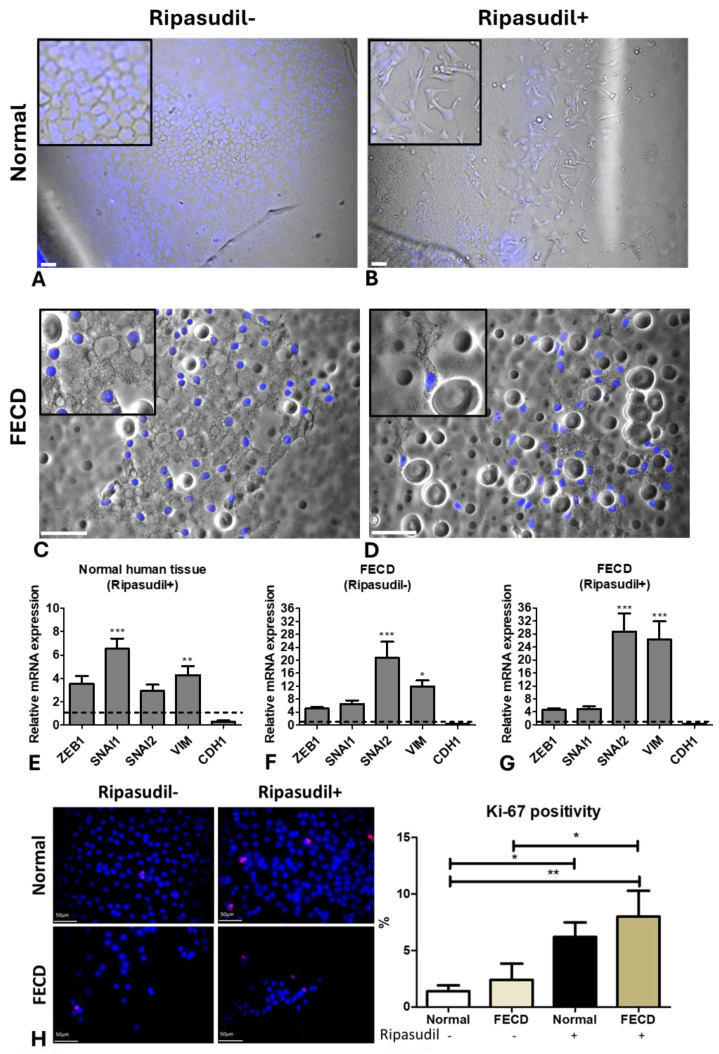
Ex vivo normal and FECD tissue. Normal corneal tissue stained with Hoechst showing (**A**) a hexagonal morphology without ripasudil and (**B**) an EMT-like morphology with ripasudil at the edge of the migrating cells. FECD tissue stained with Hoechst showing (**C**) rosette-like structures without ripasudil and (**D**) an EMT-like morphology with ripasudil. RT-PCR analysis of EMT genes in (**E**) normal tissue treated with ripasudil, and FECD tissue (**F**) untreated and (**G**) treated with ripasudil, normalized to untreated normal tissue. (**H**) The expression and analysis of the Ki-67-positive cell percentage on normal and FECD tissues and those treated with ripasudil. Blue: nucleus (Hoechst+); Red: proliferating cells (Ki67+). * *p* < 0.05; ** *p* < 0.01; *** *p* < 0.001. Figure insert—high magnification. The data are expressed as mean ± SEM. Scale: 50 µm.

Although we trypsinized the cells from normal human cadaveric donor tissues to investigate the effect of ripasudil on individual cell velocity and displacement, the source of peripheral cells in the DSO is usually present in the form of an intact monolayer. Next, we sought to investigate the migration of HCEnCs without trypsin to establish the migration pattern and phenotype of these cells in response to ripasudil by the live-cell imaging technique. We observed that normal peripheral cells migrate as a confluent sheet onto their native DMs without ripasudil treatment, likely mimicking the cellular response to DSO (Figure 7A, Supplementary Video S1). However, the sheet of cells that was exposed to ripasudil (without trypsin) showed individual cells breaking from the monolayer at the edge and assuming a spindle-shaped morphology, enabling a more widespread individual cellular migration onto the bare DMs (Figure 7B; Supplementary Video S2). A similar phenomenon was observed in the FECD cells treated without (Figure 7C) and with ripasudil (Figure 7D). This cellular behavior may be conducive to the increased migration leading to a higher rate of wound healing observed in patient case studies treated with ripasudil.

To investigate what phenotype the cells assumed during the enhanced migration, we performed RT-PCR after the ripasudil treatment, comparing the normal and FECD cells on native DMs to the untreated controls. The normal human donor tissues showed the upregulation of *SNAI1* (6.5-fold; *p* < 0.001) and *VIM* (4.3-fold; *p* < 0.01; Figure 7E) when treated with ripasudil. The FECD cells showed an upregulation of *SNAI2* (20.7-fold; *p* < 0.001) and *VIM* (11.9-fold; *p* < 0.05; Figure 7F) compared to normal tissues without the ripasudil treatment, similar to previous studies [47,48]. Furthermore, the FECD tissues treated with ripasudil showed even further upregulation of *SNAI2* (28.7-fold; *p* < 0.001) and *VIM* (26.3-fold; *p* < 0.001) (Figure 7G) when compared to untreated normal controls. *CDH1* was downregulated after the ripasudil treatment in all the tissues, indicating the loss of tight junctions.

To investigate whether, in addition to enhancing migration, ripasudil causes cellular proliferation, the normal and the FECD donor tissues were stained with a Ki-67 marker. Ki-67 is present in the active phases of the cell cycle and its expression increases during cell cycle progression; hence, it is often used as a proliferative marker. We found no difference in percentage of Ki-67-positive cells between the normal (1.4 ± 1.1%) and FECD (2.4 ± 3.2%) groups (*p* > 0.05). However, an increased Ki-67 percentage positivity was observed in the normal (6.2 ± 2.9%; *p* < 0.05) and FECD tissues (8.0 ± 5.1%; *p* < 0.05) after the ripasudil treatment for 24 h (Figure 7H), leading to a 4.4-fold and 3.3-fold increase in Ki-67 expression in the normal and FECD tissues, respectively, compared to the untreated controls, with no difference in fold increase between the normal and FECD cells.

### 3.8. Ex Vivo DSO Model Using Donor DMEK Tissues and SMILE Lenticules

**Figure 8:**
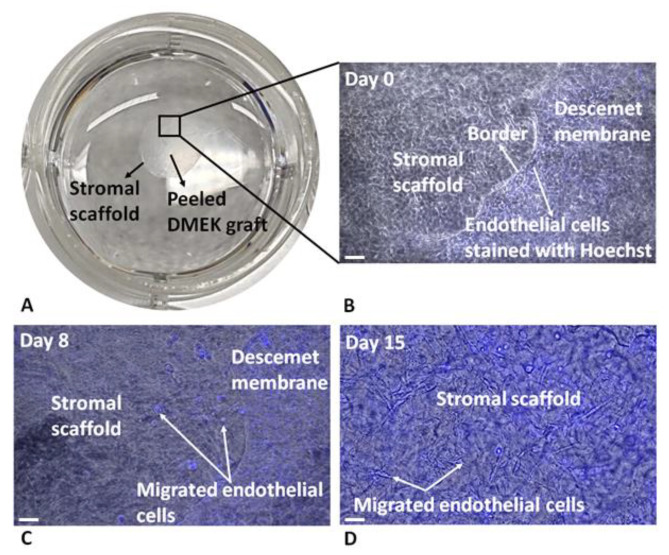
Ex vivo Descemet’s Stripping Only model. (**A**) Ex vivo DSO model as observed macroscopically in a 12-well cell culture plate. (**B**) Microscopic image of the DM attached on the stroma (SMILE lenticule) with stained endothelial cells at day 0. (**C**) Image of migrating endothelial cells from the DM to the stroma at day 8 and (**D**) migrated cells on the stroma at day 15. Scale: 50 µm.

Since endothelial cells migrate straight onto the stroma during DSO in vivo, we developed an ex vivo DSO model using SMILE lenticules. Peeled and stained DMEK tissues obtained from normal human cadaveric donors were draped over the SMILE lenticules/stromal substrates (Figure 8A). In addition, to investigate the effect of the modified ECM on cell migration, we coated SMILE lenticules with FNC and analyzed the velocity and displacement of normal HCEnCs with and without ripasudil. The representative live cell images of Hoechst-stained endothelial cells show gradual HCEnC migration from the DM to the stroma at day 8 and day 15, allowing for the imaging of the cellular morphology and the quantification of the migration velocity and displacement of cells traveling from the DM to the stroma (Figure 8B–D).

**Figure 9:**
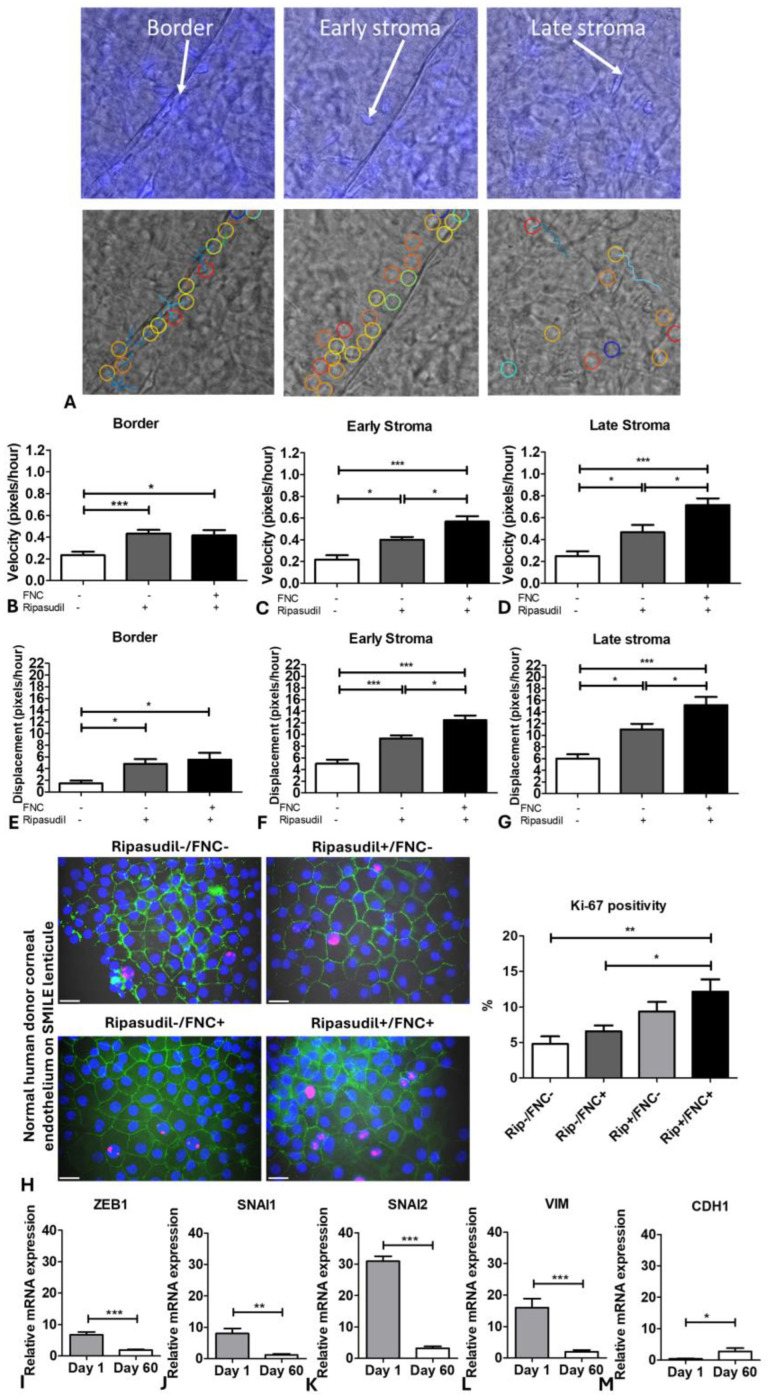
Velocity and displacement of normal corneal endothelial cells from the Descemet membrane to the stroma (SMILE lenticule) using ex vivo Descemet’s Stripping Only model. (**A**) Trackmate analysis of three regions of interest to analyze the velocity and displacement of cells on the stroma with and without FNC/ripasudil. Velocity of cells at (**B**) the border, (**C**) early stroma, and (**D**) late stroma, and displacement of cells at (**E**) the border, (**F**) early, and (**G**) late stroma with FNC (+/−) and ripasudil (+/−). (**H**) Expression of ZO-1 marker and Ki-67 positivity on day 60. EMT genes (**I**) *ZEB1*, (**J**) *SNAI1*, (**K**) *SNAI2*, (**L**) *VIM*, and (**M**) *CDH1* expressed by normal corneal endothelial cells migrated on the stroma after day 1 and day 60 of storage. Blue: nucleus (Hoechst+); Red: proliferating cells (Ki67+); Green: intercellular tight junctions (zonula occludens 1 (ZO-1+)). * *p* < 0.05; ** *p* < 0.01; *** *p* < 0.001. The data are expressed as mean ± SEM. Scale: 20µm.

The cell migration was regionally analyzed at (a) the border on the DM, (b) the early stroma (on the SMILE lenticule but just outside the border of DM), and c) the late stroma (on the SMILE lenticule away from the DM) using Trackmate analysis (Figure 9A). Treatment with FNC alone did not result in a significant increase in velocity or displacement (Supplementary Figure S5). However, a significant increase in velocity (2.1-fold; *p* < 0.001) was observed when the cells were treated with ripasudil or a combination of ripasudil and FNC (2.0-fold; *p* < 0.05) at the border compared to the untreated (ripasudil-/FNC-) tissues (Figure 9B). A similar trend of increased velocity was observed at the early stroma when the cells were treated with ripasudil alone (2.4-fold; *p* < 0.05) and concurrent ripasudil and FNC treatment (3.0-fold; *p* < 0.001). Interestingly, a 1.5-fold increase (*p* < 0.05) in velocity was observed when the tissues were treated with ripasudil and FNC compared to the treatment with ripasudil alone (Figure 9C). The cells treated with ripasudil (2.3-fold; *p* < 0.05) and the concurrent ripasudil and FNC treatment (3.4-fold; *p* < 0.001) showed accelerated velocity compared to the untreated donors at the late stroma. A 1.5-fold increase (*p* < 0.05) in velocity at the late stroma was observed when the tissues were treated with ripasudil and FNC compared to the treatment with ripasudil only (Figure 9D).

Furthermore, the cells at the border showed a significant increase in displacement (2-fold; *p* < 0.05) when treated with ripasudil and concurrent treatment with ripasudil and FNC (2.5-fold; *p* < 0.05) compared to the untreated controls (Figure 9E). At the early stroma, ripasudil (1.9-fold; *p* < 0.001) and ripasudil with FNC (2.5-fold; *p* < 0.001)-treated cells showed a higher displacement compared with the untreated controls, with a 1.4-fold increase (*p* < 0.05) in displacement observed between ripasudil alone and concurrent treatment with ripasudil and FNC (Figure 9F). Similarly, cells showed a significantly higher displacement at the late stroma with ripasudil (2.1-fold; *p* < 0.05) and concurrent treatment of ripasudil with FNC (2.7-fold; *p* < 0.001) compared to the untreated controls. Moreover, a 1.5-fold increase in the displacement at the late stroma (*p* < 0.05) was observed when the cells were treated with ripasudil and FNC compared to ripasudil alone (Figure 9G). Application of FNC to the SMILE lenticules increased the velocity and displacement of cells on the stroma compared to the border, indicating that this common extracellular matrix component of basal lamina facilitates cellular migration in the absence of the natural DM basement membrane.

The ex vivo DSO lenticules were stored for 60 days in the culture medium, and then assayed for stromal re-endothelialization and cellular proliferation. Expression of ZO-1 staining indicated the formation of a monolayer with tight junctions at the late stroma in all of the lenticules. A significantly higher percentage of Ki-67-positive cells was observed in the groups treated with ripasudil without FNC (9.4 ± 2.9%; *p* < 0.05) and ripasudil with FNC (12.2 ± 3.8%; *p* < 0.01) compared with the untreated controls (4.8 ± 2.4%) (Figure 9H). The cells treated with only FNC (without ripasudil) did not show a significantly higher Ki-67 percentage positivity (6.6 ± 3.8%) compared to the untreated controls. However, the cells treated with ripasudil alone and a combination of ripasudil with FNC showed a higher percentage of Ki-67 positivity, indicating that ripasudil contributes towards proliferation.

Although the EMT markers *SNAI2* (31-fold; *p* < 0.001) and *VIM* (16-fold; *p* < 0.001) were upregulated at day 1, they showed a significant reduction at day 60 after the cells were treated with a combination of ripasudil and FNC. A reduction in EMT markers *ZEB1* (3.5-fold; *p* < 0.001; Figure 9I), SNAI (6.7-fold; *p* < 0.01; Figure 9J), *SNAI2* (9.5-fold; *p* < 0.001; Figure 9K), and *VIM* (8.2-fold; *p* < 0.001; Figure 9L) with the upregulation of *CDH1* (6.7-fold; *p* < 0.05; Figure 9M) was observed at day 60 compared to day 1 of the culture period, indicating that the cells exit the EMT phase required for migration. Normal corneal endothelial cell migration on the stroma (FNC+/−) treated with ripasudil (+/−) is shown in Supplementary Videos S3–S11.

## 4. Discussion

Descemet Stripping Only (DSO), a relatively new and innovative corneal surgical procedure, involves the selective removal of damaged endothelium and DM from a central circular area on the posterior corneal surface. Unlike traditional treatment methods that rely on donor corneal tissue transplantation, DSO prompts the migration of the patient’s own peripheral endothelial cells to migrate, redistribute, and restore normal corneal function. This approach, by utilizing endogenous cells rather than cadaveric donor tissue, contributes to a reduction in the global demand for human donor tissues. Clinical investigations have validated the efficacy of DSO [17,18,19,20]. Furthermore, the use of topical ripasudil has been advocated as a potential adjunctive treatment for FECD in conjunction with DSO [20] and has been shown as a reliable intervention with an acceptable safety profile for the selected FECD patient group [49]. However, due to the unpredictable clinical outcomes following DSO, our study aimed to elucidate the mechanistic aspects of cell migration using immortalized cells, normal and FECD cells on their native DMs, and a novel ex vivo DSO model closely mimicking the migration of HCEnCs in vivo. Additionally, we explored the impact of ripasudil, a Rho kinase inhibitor, and the potential of ECM modification in facilitating cell migration to potentially reduce the rehabilitation time and enhance the efficiency of DSO to reduce the overall demand of donor corneas.

Previous investigations have explored the phenomenon of cell migration utilizing various in vitro and ex vivo models. Pipparelli et al. conducted a study wherein they observed an expedited in vitro wound closure through a scratch assay and increased proliferation of HCEnCs when subjected to ROCK inhibitor (Y-27632) treatment [30]. This aligns with our findings, where we also observed enhanced cell migration ex vivo with ripasudil. In addition, Miron et al. conducted a study showcasing cell migration from the DM to the stroma of the same tissue, primarily through collective cell migration leading to the formation of a cell monolayer devoid of the DM substrate, both with and without the ROCK inhibitor [50]. Ex vivo models involving scratch or peel techniques have been employed to assess the potential of residual CEnCs from normal human cadaveric donor tissues to regenerate on its native DM lacking endothelial cells [51]. Our study employed ex vivo models to investigate cellular migration dynamics, capturing the real-time velocity and displacement of cells. While some previous studies focused on collective cell migration or used specific inhibitors, our study uniquely combined models studying cells as monolayers and individual cells. This approach provides a broader understanding of migration mechanisms. Earlier studies have focused on inhibitors alone, whereas our study also explored the effect of an FNC coating in addition to ripasudil treatment. This dual-modulation approach showed how microenvironmental factors influence cellular migration compared to studies focusing solely on pharmacological interventions, which has significant clinical implications, especially in reducing the visual rehabilitation time after DSO treatment.

Previous studies have shown that FECD cells have a higher migration speed compared to normal cells [38]. To elucidate the mechanism of heightened cell migration in FECD, we explored the drive for cellular cytoskeleton assembly in both normal and FECD cell lines and detected that FECD cells exhibit the higher activation of Rac1 compared to normal cells at baseline; however, these findings were further enhanced with the use of the RhoA inhibitor. Such enhanced migratory capability might be attributed to the antagonistic relationship between RhoA and Rac1, where the activation of one GTPase leads to the inhibition of the other [52]. Herein, we show that the inhibition of RhoA with ripasudil resulted in the activation of Rac1 and cell migration via membrane protrusion. Cell migration involves the dynamic reorganization of the actin cytoskeleton [44,45,46], particularly during lamellipodia formation. Similarly, our study detected actin polymerization into lamellipodia at the leading edge of the migrating cells, which was enhanced with the supplementation with a selective ROCK inhibitor. Overall, we showed that the inhibition of RhoA leads to the activation of Rac1, which, in turn, activates the WAVE regulatory complex and stimulates the ARP2/3 complex [43,44,45,46]. This process generates filaments exerting protrusive force against the cell membrane, while the overall actin network undergoes retrograde flow due to contractile forces at the cell lamella, contributing to lamellipodial protrusion [36,37]. Ripasudil, by inhibiting RhoA and activating Rac1, thus facilitates migration, resulting in increased lamellipodial protrusion persistence, which was further confirmed by immunostaining with the ARPC2 marker.

In FECD, the upregulation of EMT has been shown as a reactive event in response to pathological stress, including oxidative stress, which generates reactive oxygen species and causes the production of excessive collagen-rich ECM, followed by an altered HCEnC morphology [47]. Previously, we have shown the upregulation of the EMT markers *SNAI1*, a known regulator of EMT in neural crest cell migration [53], N-Cadherin, and *ZEB1* in FECD [47]. Similarly, ripasudil treatment led to the upregulation of *SNAI2*, *VIM*, and *ZEB1*, along with the repression of E-cadherin, correlating with the time of increased cellular migration. Since the Rac1 signaling pathway has also been implicated in inducing the EMT phenotype, elevated levels in response to ripasudil indicate the activation of the mesenchymal state, necessary for the reorganization of the cytoskeleton during the migration [52,54].

A typical characteristic of FECD is the excessive loss of cells that results in improper maintenance of the barrier function [22,55]. The loss of cells leaves a void area on the DM. This stimulates the surrounding cells at the wound site to respond through cell enlargement, monolayer spreading, or individual cell migration. Since the prevailing thought is that HCEnCs have a limited proliferative capacity, the primary response of the neighboring cells is thought to constitute a combination of cell spreading or migration leading to cell enlargement and membrane ruffling at the wound edge, as observed by lamellipodia formation [56]. This phenomenon is also observed clinically, where age-related polymorphisms can be seen using specular microscopy [57]. However, following a larger wound, along with cell enlargement, the coordinated movement of the surrounding cells that are contracted and pulled to close the wound is also observed in the monolayer spreading of cells [58,59,60]. Successful regeneration of the central corneal endothelium after DSO depends on the cellular migration of peripheral HCEnCs, highlighting the importance of the wound-repair mechanism. Similarly, the concept of possible cell proliferation has been challenged by showing a significant decrease of 10% to 40% in the peripheral endothelial cell counts after 12-15 months, which suggests a primary promigratory role of peripheral HCEnCs in the clinical setting [16,20]. However, in our study, an increase in a proliferative marker (Ki-67) was observed after treating the cells with ripasudil, which indicates that, although the primary action of wound healing could be migration, some cells undergo cell division, increasing the number of endothelial cells associated with wound closure.

One of the crucial factors for normal HCEnC migration is the presence of an intact DM, as it maintains the ECM microenvironment and facilitates migration compared to the bare stroma [51]. The speed (velocity) and distance covered (displacement) can be influenced by several factors, including the presence of DMs or supplementation with a ROCK inhibitor [51,61]. Using the ex vivo DSO model, we observed that the cells migrated on the bare DM faster than they migrated on the bare stroma. However, when the ECM microenvironment was modified with an FNC coating, the rate of migration improved and increased further with the ripasudil treatment. The ECM and its microenvironment is significantly important to increase the migration rate, which would reduce the wound-healing time [62,63]. In addition, migration speed differences have also been reported when normal HCEnCs were seeded on normal-DM or FECD-DM, indicating the capacity of the ECM to affect the corneal endothelial cell behavior [64]. Ong Tone et al. described that FECD cells display an increased migration speed compared with normal cells when placed on the same substrate [38]. This led to a hypothesis that FECD cells have intrinsic promigratory changes due to Rac1 activation and EMT induction, in addition to potential ECM changes, that contribute to an increased migratory phenotype. Interestingly, we also observed the downregulation of EMT genes once the cells induced contact inhibition and formed a monolayer with tight-junction proteins (expression of ZO-1 marker). The eventual loss of the EMT phenotype is an important phenomenon for maintaining the endothelial cells as a monolayer, further allowing for an active pumping mechanism.

Although we provide evidence that ripasudil enhances migration in FECD, our study has several limitations. The study was limited by use of only several cell lines, which did not provide a sufficient sample size to explore the effect of sex and genetic background on the cellular migration. Since FECD is a female-predominant disease, it is essential to study the role of sex hormone receptors on cellular migration in the future. In addition, we were not able to genotype the FECD or normal donors to identify whether there is a differential response to ripasudil depending on the genetic makeup of the tissues. In a recent publication, Yan J et al. documented that TCF4-B promotes enhanced cellular migration through the modulation of microtubule dynamics in FECD, independently of EMT processes. Moreover, the study underscored the dysregulation of microtubule stability in FECD [65]. Thus, deciphering genetic variations and identifying specific genetic profiles could potentially aid in patient selection for DSO procedures, ultimately aiming to minimize recovery times in the future.

In summary, our study not only elucidates the underlying mechanisms of enhanced cell migration in FECD but also highlights a potential strategy to optimize the clinical application of DSO. The combination of ripasudil and ECM modification presents a promising avenue for enhancing the efficiency of CEnC therapy, ultimately benefiting patients by improving surgical outcomes and addressing the global challenge of corneal donor shortage. Future research should continue to explore these avenues to refine treatment protocols and broaden the impact of cell migration specially in DSO.

## 5. Conclusions

In summary, our findings demonstrate that FECD cells exhibit an accelerated migratory response, likely due to EMT, causing an enhanced wound-healing rate. This phenomenon is attributed to the activation of Rac1, leading to the formation of lamellipodia and membrane ruffles. While ripasudil demonstrates a potential to augment proliferation, our observations underscore that migration is the primary mechanism in the wound-healing process of FECD, especially considering that the proliferative cells constitute less than 10% of the population. Despite ripasudil’s capacity to induce migration, we posit that ECM modification could further amplify the migratory behavior of HCEnCs in FECD patients. The promigratory effects of ripasudil and concurrent treatment with FNC hold promise in minimizing the rehabilitation time post-DSO and alleviating the global demand for human donor corneas by increasing the utilization of DSO.

## Figures and Tables

**Table 1:** Donor characteristics of the normal human cadaveric tissues and FECD specimen.

	Normal (n = 43)	FECD (n = 16)
**Age (years)**	59 ± 6	73 ± 8
**Sex (M/F)**	19/24	3/13
**Preservation time (average ± SD)**	5.8 ± 1.3 (days)	4.5 ± 1.4 (days)
**ECD (cells/mm^2^)**	2370 ± 431	N/A

**Table 2:** Sample size of the normal human cadaveric tissues and FECD specimen used for ex vivo cell migration and DSO studies.

**Ex Vivo Random Migration**			
**Treatment**	**Analysis**	**Donor Tissue**	**FECD Specimen**
**Ripasudil- (n = 8)**	Live-cell imaging	n = 8	n = 8
	RT-PCR (EMT genes)	n = 4	n = 4
	Immunostaining (Ki-67)	n = 4	n = 4
**Ripasudil+ (n = 8)**	Live-cell imaging	n = 8	n = 8
	RT-PCR (EMT genes)	n = 4	n = 4
	Immunostaining (Ki-67)	n = 4	n = 4

**Ex Vivo DSO Model (Migration of Cells from DM to Stroma)**			
**Treatment**	**Analysis**	**Donor Tissue**	**FECD Specimen**
**Ripasudil-/FNC- (n = 6)**	Live-cell imaging	n = 3	N/A
	Immunostaining (ZO1 + Ki-67)	n = 3	
**Ripasudil+/FNC- (n = 6)**	Live-cell imaging	n = 3	
	Immunostaining (ZO1 + Ki-67)	n = 3	
**Ripasudil-/FNC+ (n = 6)**	Live-cell imaging	n = 3	
	Immunostaining (ZO1 + Ki-67)	n = 3	
**Ripasudil+/FNC+ (n = 9)**	Live-cell imaging	n = 3	
	Immunostaining (ZO1 + Ki-67)	n = 3	
	RT-PCR (EMT genes)	n = 3	

## Data Availability

The raw data supporting the conclusions of this article will be made available by the authors on request.

## Supplementary Materials

The following supporting information can be downloaded at: https://www.mdpi.com/article/10.3390/cells13141218/s1. Figure S1. (**A**) Viability analysis using the CellTiter-Glo 2.0 assay to determine the number of viable cells after treating the cells with different concentrations of ripasudil. (**B**) Toxicity analysis using ethidium homodimer at different concentrations of ripasudil. Blue: nucleus (Hoechst+); Red: dead cells (ethidium homodimer+ indicated by white arrows). * *p* < 0.05; ** *p* < 0.01; *** *p* < 0.001. The data are expressed as mean ± SEM. Scale: (**A**) 100 µm; (**B**) 250 µm. Figure S2: Baseline (**A**) velocity and (**B**) displacement of normal and FECD cells without the treatment with ripasudil. Fold change in (**C**) velocity and (**D**) displacement of cells treated with ripasudil compared to their respective untreated controls. ** *p* < 0.01; *** *p* < 0.001. The data are expressed as mean ± SEM. Figure S3. Wound closure of all of the studied cell lines (normal and FECD) at baseline (without ripasudil treatment). ** *p* < 0.01; *** *p* < 0.001. Figure S4. (**A**) Rac1 activation in normal and FECD cells after ripasudil treatment and (**B**) Pearson’s correlation between normal and FECD cells after ripasudil treatment. * *p* < 0.05; ** *p* < 0.01; *** *p* < 0.001. The data are expressed as mean ± SEM. Figure S5. Velocity and displacement of cells at the border, early stroma, and late stroma with and without FNC treatment. ns = not significant. Video S1. Normal human corneal endothelial cells migrating as a confluent sheet onto their native Descemet’s membrane without ripasudil treatment. Video S2. Individual cells breaking from the monolayer at the edge enabling individual cell migration onto the bare Descemet’s membrane. Video S3. Normal human corneal endothelial cells migrating at the border between the Descemet’s membrane and the stroma without FNC and ripasudil treatment. Video S4. Normal human corneal endothelial cells migrating at the early stroma without FNC and ripasudil treatment. Video S5. Normal human corneal endothelial cells migrating at the late stroma without FNC and ripasudil treatment. Video S6. Normal human corneal endothelial cells migrating at the border between the Descemet’s membrane and the stroma without FNC with ripasudil treatment. Video S7. Normal human corneal endothelial cells migrating at the early stroma without FNC with ripasudil treatment. Video S8. Normal human corneal endothelial cells migrating at the late stroma without FNC with ripasudil treatment. Video S9. Normal human corneal endothelial cells migrating at the border between the Descemet’s membrane and the stroma with FNC and ripasudil treatment. Video S10. Normal human corneal endothelial cells migrating at the early stroma with FNC and ripasudil treatment. Video S11. Normal human corneal endothelial cells migrating at the late stroma with FNC and ripasudil treatment.
