# Supplementary material for: Enhanced Migration of Fuchs Corneal Endothelial Cells by Rho Kinase Inhibition: A Novel Ex Vivo Descemet’s Stripping Only Model
Source: Cells. 2024 Jul 19;13(14):1218. doi: 10.3390/cells13141218 (PMC11274477; doi:10.3390/cells13141218)
Supplement: Supplementary file 1 [file cells-13-01218-s001.zip › cells-3067809-supplementary.pdf]

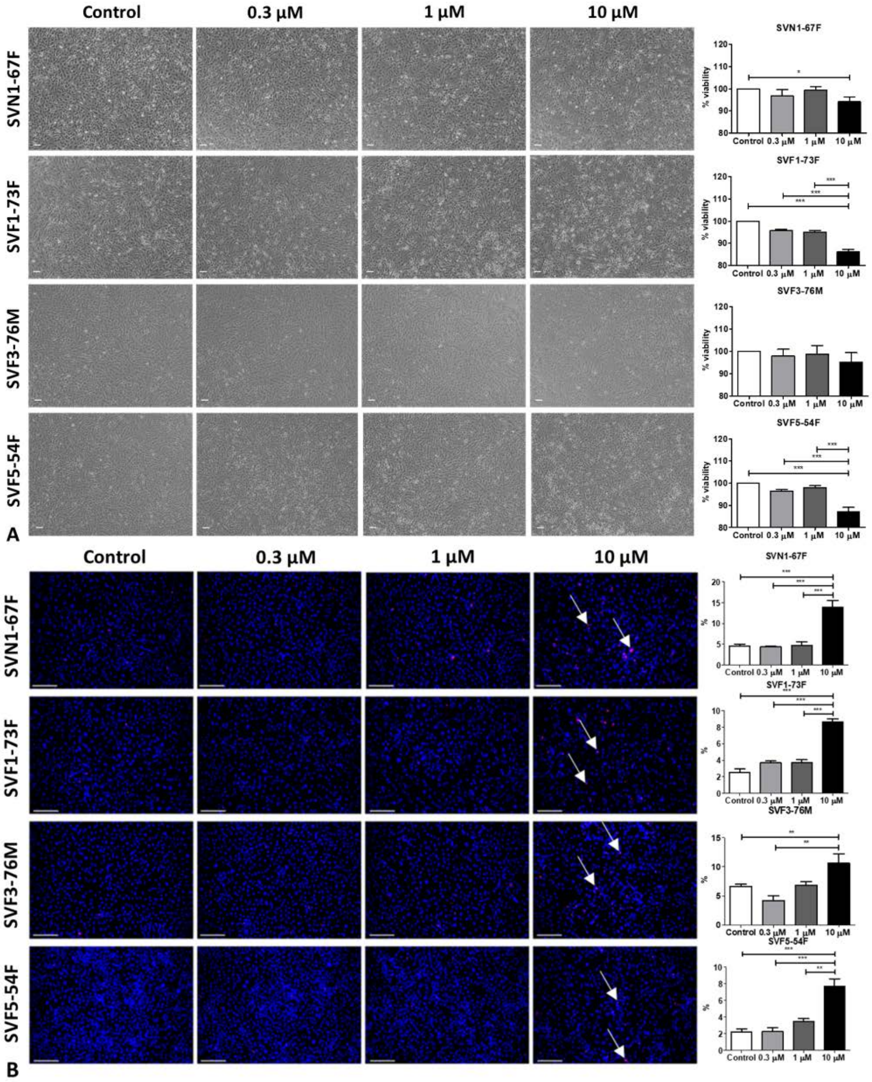


Supplementary Figure S1A and S1B.

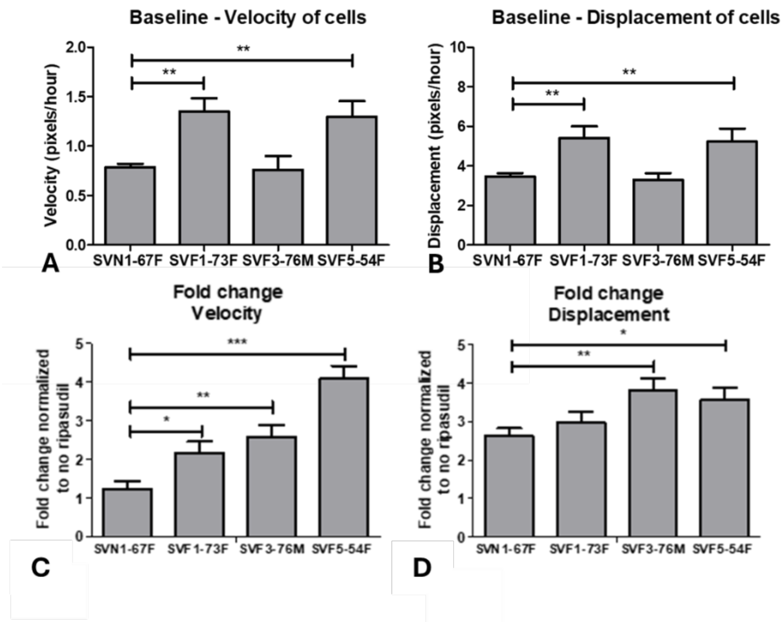


Supplementary Figure S2A, S2B, S2C and S2D

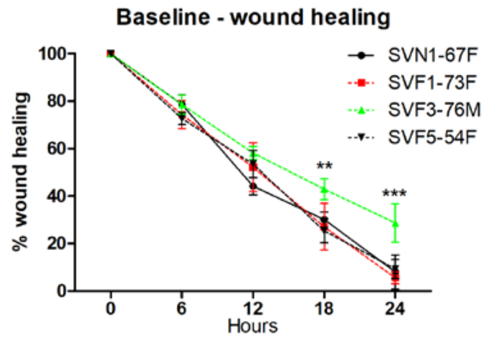

Supplementary Figure S3

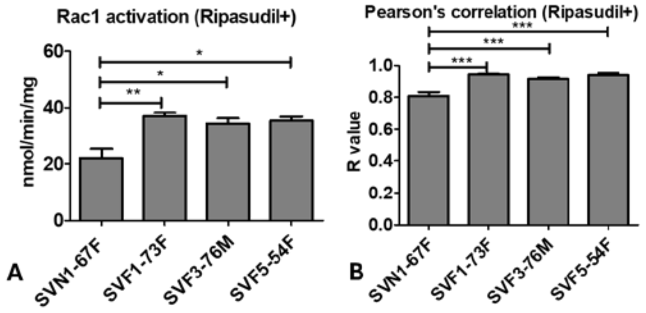

Supplementary Figure S4A, S4B

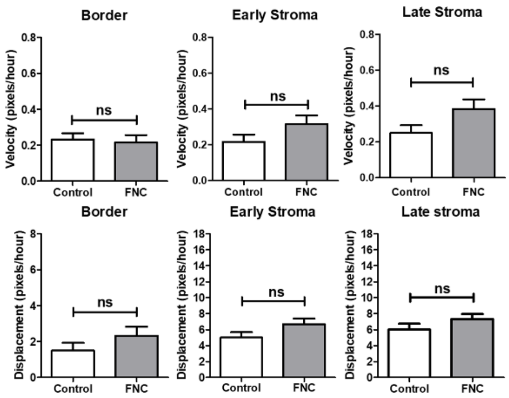

Supplementary Figure S5
